# Significance of Anti-Nuclear Antibodies and Cryoglobulins in Patients with Acute and Chronic HEV Infection

**DOI:** 10.3390/pathogens9090755

**Published:** 2020-09-16

**Authors:** Thomas Horvatits, Julian Schulze zur Wiesch, Susanne Polywka, Gustav Buescher, Marc Lütgehetmann, Elaine Hussey, Karoline Horvatits, Sven Peine, Friedrich Haag, Marylyn M. Addo, Ansgar W. Lohse, Christina Weiler-Normann, Sven Pischke

**Affiliations:** 1 I. Department of Medicine, Gastroenterology and Hepatology, with the Sections Infectious Diseases and Tropical Medicine, University Medical Center Hamburg-Eppendorf, 20246 Hamburg, Germany; j.schulze-zur-wiesch@uke.de (J.S.z.W.); g.buescher@uke.de (G.B.); e.hussey@uke.de (E.H.); k.horvatits@uke.de (K.H.); m.addo@uke.de (M.M.A.); alohse@uke.de (A.W.L.); cweiler@uke.de (C.W.-N.); s.pischke@uke.de (S.P.); 2 German Center for Infection Research (DZIF), Hamburg-Lübeck-Borstel and Heidelberg Partner sites, 20359 Hamburg, Germany; mluetgehetmann@uke.de; 3 Institute of Microbiology, Virology and Hygiene, University Medical Center Hamburg-Eppendorf, 20246 Hamburg, Germany; polywka@uke.de; 4 Institute of Transfusion Medicine, University Medical Centre Hamburg-Eppendorf, 20246 Hamburg, Germany; s.peine@uke.de; 5 Institute of Immunology, University Medical Center Hamburg-Eppendorf, 20246 Hamburg, Germany; haag@uke.de; 6 Martin Zeitz Center for rare diseases, University Medical Center Hamburg-Eppendorf, 20246 Hamburg, Germany

**Keywords:** hepatitis E, HEV, autoimmune response, antibodies, cryoglobulins

## Abstract

Background: Hepatitis E virus (HEV) has been associated with immunological phenomena. Their clinical significance, however, still needs to be clarified, that is, whether cryoglobulins or autoantibodies impact overt disease in HEV-infected individuals. To better understand, we analyzed these different immune phenomena in three cohorts, each representing different types of HEV infection. Methods: The cohorts included: (i) immunocompetent patients with acute hepatitis E, (ii) immunosuppressed patients with chronic hepatitis E, and (iii) individuals with asymptomatic HEV infection. Together, they consisted of 57 individuals and were studied retrospectively for the presence of anti-nuclear antibodies (ANAs), cryoglobulins, and serum total IgG. They were then compared with a control cohort of 17 untreated patients with chronic hepatitis B virus (HBV) infection or hepatitis C virus (HCV) infection. Results: Thirteen (23%) were immunocompetent patients with acute hepatitis E (median alanine aminotransferase (ALT) = 872 U/L), 15 (26%) were immunosuppressed patients with chronic hepatitis E (median ALT = 137 U/L), and 29 (51%) were blood donors with asymptomatic HEV infection (median ALT = 35 U/L). Overall, 24% tested positive for elevated ANA titers of >1:160, and 11% presented with a specific ANA pattern. ANA detection was not associated with the type of HEV infection, IgG levels, sex, or age. All individuals tested negative for anti-mitochondrial antibodies, anti-neutrophil cytoplasmic antibodies, liver-kidney microsomal antibodies, anti-myeloperoxidase-, and anti-proteinase-3 antibodies. Five patients (9%) tested positive for cryoglobulins. Notably, cryoglobulinemia was present in overt hepatitis E (Groups (i) and (ii); one acute and four chronic HEV infections), but was not present in any of the asymptomatic blood donors (*p* = 0.02). The frequency of cryoglobulins and elevated ANAs did not differ significantly between HEV and HBV/HCV patients. Conclusion: In line with findings on HBV and HCV infections, we frequently observed detection of ANAs (24%) and cryoglobulins (9%) in association with HEV infections. The presence of cryoglobulins was limited to patients with overt hepatitis E. We add to the findings on the immune phenomena of hepatitis E.

## 1. Introduction

Hepatitis E virus (HEV) is a pathogen occurring worldwide and it can lead to hepatitis, an inflammatory liver disease [1]. While the large majority of individuals infected with HEV experience an asymptomatic course with spontaneous viral clearance, a small group of infected individuals develop clinically overt hepatitis E, resulting in elevated liver enzymes and sometimes in jaundice. Furthermore, immunocompromised individuals with HEV infection are at risk of developing chronic hepatitis E, leading to fibrosis, cirrhosis, and associated complications [2,3,4]. Recent data indicate that symptomatic HEV infection is not limited to the liver; it can also affect other organs. For example, it can affect the pancreas, leading to pancreatitis and the nervous system, leading to neuralgic amyotrophy, Guillain-Barré syndrome, and other problems [5,6,7,8,9,10].

The presence of autoantibodies has been described in HEV infection and in other viral diseases [11,12,13]. In contrast to true autoimmune diseases, there has been debate on the role of the immunological bystanders or the non-specific phenomena in HEV. There are also conflicting data related to autoimmune hepatitis (AIH) [14,15]. An increased proportion of HEV-seropositive patients was described in two cohorts of German and Austrian patients with AIH compared with healthy controls [14,15]. This suggested HEV as a possible trigger for the development of AIH, but a large Dutch cohort showed similar proportions of seropositive results in AIH patients compared with controls [16]. Whether or not the higher rate of seropositivity in AIH patients is merely due to false-positive testing because of higher levels of immunoglobulins has been debated [13]. In general, the presence of autoantibodies in sera from patients with active HEV infection is common [12,17]. This may result in a false AIH diagnosis when the underlying HEV infection is overlooked, and it may also result in unsuitable immunosuppressive treatment being used [12,13,15,16,18,19,20,21,22,23,24,25,26].

Other immune phenomena such as cryoglobulinemia, have also been linked to HEV infection [27,28,29]. Cryoglobulinemia is defined as the presence of immunoglobulins, which precipitate at temperatures below 37 °C and re-dissolve after warming up again [30]. It is important to differentiate between the laboratory phenomena of cryoglobulinemia and the clinical disease associated with cryoglobulinemia, often characterized by vasculitis-associated symptoms [31]. Type I cryoglobulinemia is defined by the presence of one monoclonal subtype of immunoglobulins and is associated with malignant disease. Type II and type III cryoglobulinemia, known as mixed-type cryoglobulinemia, are not only associated with autoimmune diseases, but also with different viral diseases, such as the hepatitis C virus (HCV) infection, in addition to hepatitis A and B [30,32,33]. A case report described the appearance of cryoglobulinemia in a liver transplant recipient [27]. In line with this, there are other recent reports suggesting that HEV plays an essential role in inducing cryoglobulinemia: large studies on HEV-infected solid organ transplant (SOT) recipients revealed that up to 40% of kidney transplant recipients tested positive for cryoglobulins [27,29,34]. In a group of patients with essential cryoglobulinemia, a higher prevalence of anti-HEV IgG was found when compared with a group with cryoglobulinemia of a defined origin [34]. In line with these data, the presence of autoantibodies has also been associated with HEV infection. This was very much like other forms of viral hepatitis [13,30,33,35,36]. However, it has not yet been clarified whether cryoglobulins or autoantibodies have a pathogenetic impact and contribute to overt disease in HEV-infected individuals. We sought to clarify this by analyzing these different immune phenomena in individuals with different types of HEV infection: (i) immunocompetent patients with acute hepatitis E, (ii) immunosuppressed patients with chronic hepatitis E, and (iii) individuals with asymptomatic HEV infection. We also assessed a control cohort of 17 untreated patients with chronic hepatitis B virus (HBV) infection or HCV infection.

## 2. Results

**Figure 1:**
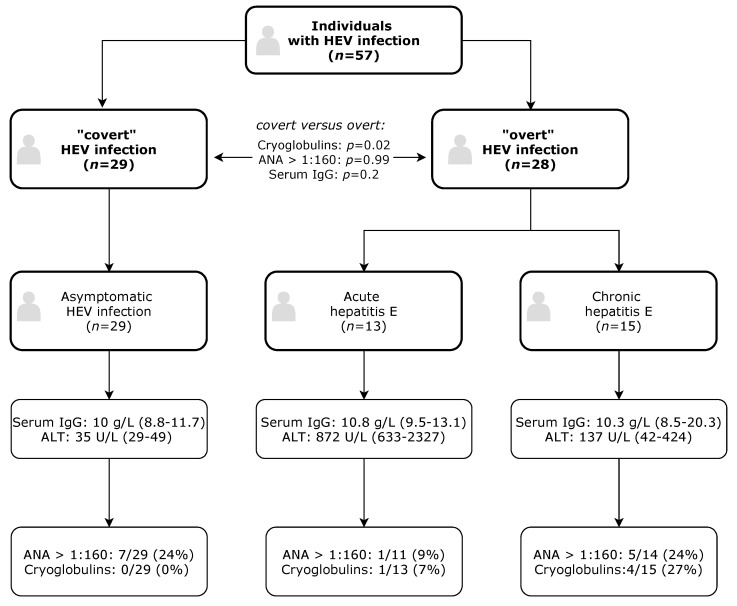
Study flow chart. Values are given as median (interquartile range (IQR)) or *n* (%). In contrast to anti-nuclear antibodies (ANAs), cryoglobulins were present in patients with overt hepatitis E (acute and chronic hepatitis E), but not in any of the covert hepatitis E virus (HEV) infections (chi-square, *p* = 0.02). Total serum IgG levels did not differ significantly between overt/covert HEV infection (Mann-Whitney-U-test, *p* = 0.2). Cryoglobulins were significantly detected more often in chronic hepatitis E, compared with acute self-limiting HEV infection (*p* = 0.014).

In this analysis, we studied the frequency of the detection of a number of autoimmune markers in 57 individuals with HEV infection. All patients were HEV-PCR-positive at the time of the study (Table 1). Thirteen (23%) immunocompetent patients presented with acute hepatitis E (median alanine aminotransferase (ALT) = 872 U/L, IQR = 633–2327 U/L), 15 (26%) immunosuppressed patients had chronic hepatitis E (median ALT = 137 U/L, IQR = 42–424 U/L, virus persistence for more than 3 months), and 29 (51%) individuals were diagnosed with asymptomatic HEV infection (median ALT = 35 U/L, IQR = 29–49 U/L) by regular testing of blood donations for HEV [37]. An overview of the study is shown in Figure 1. Aminotransferase, bilirubin, and HEV RNA levels were significantly higher in patients with overt hepatitis E (acute and chronically infected patients), compared with those with asymptomatic infection (blood donors) (*p* < 0.001; Table 1).

Anti-nuclear antibody (ANA) titers of >1:160 were observed in 13/54 (24%) of the patients, 6/54 (11%) presented with a specific ANA pattern. Titers of 1:640 were observed in seven patients (four asymptomatic, one acute, two chronic HEV patients). The most frequently observed specific ANA pattern was the homogenous nucleolar pattern (3/13, 23%), followed by the homogeneous pattern (2/13, 15%), the nuclear membrane pattern (1/13, 8%), and the dense fine speckled pattern (7/13, 54%). We could not detect a significant association of ANA elevation and type of HEV infection (overt hepatitis E vs. covert/asymptomatic HEV infection, see Figure 1), nor could we detect an association with the presence of cryoglobulins (*p* = 1), with sex (*p* = 0.49), or with age (*p* = 0.19). Serum IgG levels did not differ significantly between patients with elevated ANAs (>1:160) and those without (*p* = 0.5). We could not observe a correlation of serum IgG or IgM with HEV RNA levels (spearman’s r: −0.04, *p* = 0.76; r: 0.12, *p* = 0.52). All patients tested negative for anti-mitochondrial antibodies (AMA), anti-neutrophil cytoplasmic antibodies (ANCA), liver-kidney microsomal antibodies (LKM), and anti-myeloperoxidase-(anti-MPO)/anti-proteinase-3 (anti-PR3) antibodies. However, two asymptomatically infected blood donors with moderate titers of 1:80 and 1:320 tested positive for smooth muscle actin antibody (SMA). The two donors had ANAs, serum IgG, and liver enzymes (AST, ALT) within the normal range (<50 U/L) and a low viral load of <600 U/L. The rheumatoid factor tested positive in one patient with asymptomatic HEV infection, which also presented with an ANA titer of 1:320 at a moderate viral load of 1.600 IU/mL.

A total of 5/57 individuals with HEV infection (9%) tested positive for cryoglobulins (see Table 2). Cryoglobulinemia persisted for a median of 4 months (range 3–15 months). All of these patients were male (*p* = 0.31) and, apart from one, all were asymptomatic with regard to cryoglobulinemia. One heart transplant recipient with chronic hepatitis E developed neuralgic amyotrophy associated with measurable cryoglobulins during the course of disease. This patient remained negative for ANA, ANCA, AMA, SMA, and LKM and serum IgG was in the range of normal. Overall, cryoglobulins were only present in patients with overt hepatitis E (one patient with acute and four patients with chronic HEV infection) (*p* = 0.023). The only patient in our cohort with cryoglobulinemia associated with acute HEV infection was identified initially as a blood donor, but subsequently developed acute symptomatic hepatitis (patient #1, see Table 3). This episode of acute hepatitis E was accompanied by cryoglobulinemia and elevated serum total IgM levels (2.6 g/L).

**Figure 2:**
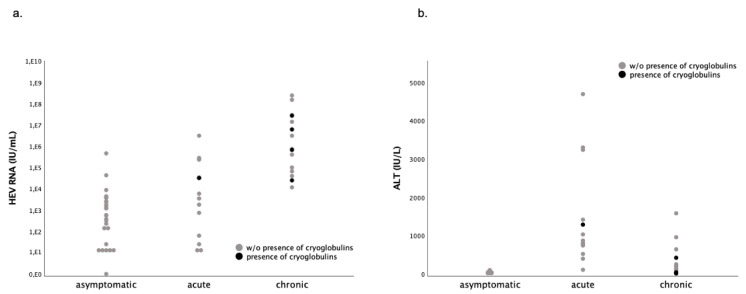
(**a**) HEV viral load in patients with asymptomatic/acute/chronic HEV infection with and without cryoglobulinemia; (**b**) alanine aminotransferase (ALT) levels in patients with asymptomatic/acute/chronic HEV infection with and without cryoglobulinemia. HEV, RNA, and ALT levels were significantly higher in patients with overt hepatitis E (acute and chronically infected patients) compared with covert/asymptomatic infection (Mann-Whitney-U-test: *p* < 0.001); in contrast, HEV RNA was significantly elevated in patients with cryoglobulinemia (Mann-Whitney-U-test: *p* = 0.018).

The presence of cryoglobulins was associated with a higher viral load (*p* = 0.018, Figure 2), but not with serum IgG levels or with liver enzymes. Interestingly, serum IgM was higher in patients with cryoglobulins (Table 2), and also in patients with overt hepatitis E (acute and chronically infected patients), compared with those with asymptomatic infection (*p* = 0.05).

Furthermore, serum creatinine levels were significantly higher in patients that tested positive for cryoglobulins compared with those that did not (median creatinine = 1.6 mg/dL, IQR = 1.3–2.8 vs. 0.9 mg/dL, IQR = 0.8–1.2; *p* = 0.01). This was most likely associated with underlying conditions, rather than HEV infection. Patients with cryoglobulins had platelets, white blood count, and lymphocyte count within the normal range. For the detailed characteristics of patients with cryoglobulins, see Table 2 and Table 3.

The presence of cryoglobulins and the elevation of ANA titers or serum IgM-levels did not differ significantly between HEV patients in comparison with a control cohort of 17 HBV or HCV infected, untreated patients (cryoglobulins: 9% versus 12%, *p* = 0.66; ANA titers >1:160: 24% versus 29%, *p* = 0.74; median serum IgM = 0.9 g/L (IQR = 0.6–1.3) versus 0.9 g/L (0.7–1.6), *p* = 0.48), whereas serum IgG levels were higher in HBV/HCV controls (median serum IgG = 10.5 g/L (IQR = 9–13.8) versus 12.3 g/L (11.4–13.6) g/L, *p* = 0.026).

## 3. Discussion

The presence of autoantibodies has been described in HEV infections and in other viral diseases [11,12,13]. Using three cohorts of patients, we studied a possible link between the type of HEV infection and the immune phenomena, such as the presence of autoantibodies and cryoglobulins. These cohorts represented different types of HEV infection and were compared with untreated HBV or HCV controls.

The main finding of our study is that the presumably non-specific antibodies, i.e., autoantibodies and cryoglobulins, frequently occur in association with HEV infections, comparable to HBV and HCV infections. The rate of ANA positivity (>1:160) in our study population was more or less comparable to previously reported data. Terziroli Beretta-Piccoli and Wu et al. reported ANA positivity rates ranging between 33% and 37% [11,12]. In contrast to our study, however, they only tested patients with acute hepatitis E, and not with chronic or asymptomatic HEV infection. In line with our data, Gilman et al. reported a rate of ANA positivity of 22% in a cohort of 1556 chronic HCV patients [35], whereas, in the German general population, the rate of ANA positivity was reported to be 33% (defined as a titer ≥1:80) [38].

Six of 54 patients (11%) showed a specific ANA pattern (“homogenous nucleolar”, “homogeneous”, and “nuclear membrane pattern”), whereas the other ANA positives showed the “dense fine speckled” pattern, which is assumed to be one of the most commonly observed indirect immunofluorescence (IIF)-ANA patterns [39]. Nevertheless, we could not observe an association of ANA occurrence or pattern with the type of HEV infection. Thus, the clinical relevance of the occurrence of ANAs in HEV-infected patients remains unknown.

Remarkably, we did not detect any association of ANAs with female gender or with age in our cohort, even though it is generally known that ANAs occur more frequently in women and in the elderly [40]. Therefore, our data support the hypothesis that the occurrence of these unspecific autoantibodies represents a para-infectious phenomenon, caused mainly by a misdirected immune response, and that it should not be seen as the initial step in the development of de novo autoimmune diseases [41,42]. This phenomenon should trigger tests to determine HEV-RNA in any patients with acute hepatitis [15,16,18], in order to prevent potential false diagnosis of AIH, due to high ANA rates among HEV infected patients. Importantly, the IgG total levels in our study did not differ between patients with chronic HEV infection and patients with acute HEV infection. However, serum IgG levels were lower in HEV patients compared with controls. Thus, our data do not indicate the possible role of enhanced IgG production as a reason for an unspecific, false positive rate of ANA testing in HEV infected patients.

Our study showed that asymptomatic HEV infection was not associated with cryoglobulinemia, but patients with overt hepatitis E (acute and chronic) were at high risk of cryoglobulinemia (9%). In addition, our data suggest that the rate of cryoglobulins seems to be comparable between the patients with HEV infection and the controls (untreated HBV/HCV patients). Of note, none of the patients presenting with cryoglobulinemia reported symptoms associated with the cryoglobulinemia disease. Our finding of cryoglobulins in HEV patients is in line with the work of others [27,29]. Unlike a previous study by the Toulouse group, our cohort is not limited to immunosuppressed individuals. Our study also included immunocompetent patients with acute hepatitis E, and asymptomatic blood donors with HEV. We compared these patients with a control cohort of untreated HBV/HCV patients. The data shed some light on the association of cryoglobulins with HEV infections. However, further studies are required to clarify the clinical relevance of this phenomenon.

The immunological processes induced by overt hepatitis E might be causative of the production of cryoglobulins, which is maintained by clonal expansions of B-cells [43]. Elevated rates of HEV infections have been observed in hematological malignancies [44,45]. Furthermore, HEV replication has very recently been demonstrated in the bone marrow [46]. However, a direct effect of HEV on B-cells and HEV replication in these cells remains to be demonstrated. Shalit et al. reported elevated serum total IgM levels in patients with hepatitis A associated cryoglobulinemia. In line with this, we found significantly elevated serum IgM levels in patients with cryoglobulins compared with those without [47]. This is a novel finding and one potential explanation could be that mixed cryoglobulins are frequently composed of large parts of IgM. However, an analysis regarding the composition of cryoglobulins was not possible in our study [48]. It is very likely that HEV induces cryoglobulinemia type II or type III in these cases, as none of the patients – even in follow-up – show signs of underlying hematological disease. Furthermore, all reported cases of HEV associated cryoglobulinemia referred to type II or type III cryoglobulins [27,28,29]. The production of cryoglobulins and ANA may rely on a misdirected B-cell response; thus, future studies should include B-cell function tests in these patients. 

In patients with hepatitis C, cryoglobulinemia has been reported to be associated with steatosis and fibrosis [49]. Furthermore, a recent study reported that even in the absence of typical cryoglobulinemia symptoms, the presence of cryoglobulins seems to activate the immune system in patients with chronic HCV infection. Low levels of cryoglobulins were associated with occurrence of rheumatoid factor and free light chains [50]. Thus, it is possible that there are silent, but clinically relevant, effects in cryoglobulinemic patients. We observed significantly elevated serum creatinine levels in patients with cryoglobulinemia, although we did not observe any documented cases of glomerulonephritis. However, elevated serum creatinine levels in immunosuppressed patients may be caused by multiple factors, such as the underlying condition, complicated post-operative course (e.g., in transplant setting), or nephrotoxicity of immunosuppressive drugs (such as calcineurin-inhibitors) [51,52]. Thus, we emphasize how important it is not to overestimate the possible association of cryoglobulinemia and elevated creatinine levels in this small sub-group (*n* = 5). However, it has been shown that patients with viral hepatitis C and the presence of cryoglobulins are at an elevated risk of developing renal failure [53]. Therefore, we suggest monitoring cryoglobulins and renal function closely in patients with chronic hepatitis E and cryoglobulinemia. Furthermore, we observed that the viral load (HEV RNA) seems to be associated with chronic hepatitis E and cryoglobulinemia, and not the degree of hepatic inflammation (serum ALT level), as illustrated in Figure 2a,b.

Due to the single center study design in a genotype 3 region, these findings may not apply to other regions and, in particular, to other genotypes. In addition, the retrospective study design means that the patient data are limited. 

In line with HBV and HCV infections, we show a strong association between HEV infection and ANA-positivity and cryoglobulins. It remains unknown whether this indicates a non-specific or potentially misdirected immune response, or whether these markers of immunity have any other function in the defense mechanisms against viral hepatitis infections. In contrast to asymptomatic HEV positive blood donors, overt hepatitis E was clearly linked to the presence of cryoglobulins and elevation of serum IgM levels. The fact that HEV infection is not merely a liver infection, but rather a systemic infection leading to a number of alterations is also underlined. Fully understanding the difference between these groups will require further studies which include human leukocyte antigen (HLA)-typing and thorough analysis of T- and B-cell responses. Our findings indicate HEV infections as a possible trigger for a presumably non-specific immune response, such as the occurrence of cryoglobulins. However, the underlying pathomechanisms and the impact on the clinical course of disease have not yet been fully understood. In future, the physicians treating patients with acute or chronic HEV infections should be aware of the possibility of HEV associated immunological phenomena (including cryoglobulinemia), and arrange specific testing as necessary.

## 4. Materials and Methods 

### 4.1. Patients

In this retrospective study, we reported data from 57 individuals with different types of HEV infection. Patients presented between August 2014 and November 2019. Patient characteristics were recorded, and patients were stratified according to type of HEV infection. Data on cryoglobulins, available in 57 patients, and on serum levels of total immunoglobulins, were analyzed, as were the presence of autoantibodies, available in 54 individuals. According to our internal laboratory standards, ANA titers of >1:160 were considered positive. All participating patients gave written informed consent. The study was approved by the local ethics committee (PV7049).

### 4.2. Testing for HEV and Definition of Hepatitis E

All individuals studied tested positive for HEV viremia by PCR. PCR testing was performed by a sensitive lab developed diagnostic quantitative PCR assay (lower limit of detection: 24 IU/mL) on a fully automated real time PCR system (Cobas 6800, Roche). This PCR assay targeting the conserved open reading frame (ORF) 2–3 region of the virus has been normalized to the first WHO standard [54]. 

Acute hepatitis E was defined as HEV infection with typical clinical and laboratory signs such as transaminitis (ALT > AST), jaundice, and/or abdominal pain. Chronic hepatitis E was defined as detection of HEV RNA in the serum for more than 3 months, according to the current European Association of the Study of the Liver (EASL) clinical practice guidelines on HEV [18]. Overt hepatitis E (ALT > 2 × upper limit of normal) was defined as acute or chronic hepatitis E, whereas covert HEV infection refers to asymptomatic blood donors detected via our routine HEV screening of blood donations.

### 4.3. Recruitment and Testing of a Control Cohort of HBV- or HCV-Infected Untreated Patients 

An unselected cohort of chronic HBV (*n* = 14) or HCV (*n* = 3) infected patients (without antiviral treatment) served as a control and was tested for cryoglobulins, ANA titers, and serum IgG and IgM levels.

### 4.4. Testing for Autoantibodies and Cryoglobulins

Testing for autoantibodies was performed by trained personnel blinded to clinical data in the Institute of Immunology. Hep-Cells and tissue slides (Euroimmun, Lübeck, Germany) were incubated, in accordance with the manufacturer’s description, and interpretation was performed manually using the Eurostar microscope (Euroimmun, Lübeck, Germany) [55]. Cryoglobulins were tested in serum as described in accordance with the local standard operating procedure. The results were reported qualitatively after 24 h of incubation [56].

### 4.5. Statistical Analysis

Data are shown as absolute number and percentage, or as median and interquartile range (IQR). Nominal variables were compared using the Chi-Square test, metric variables were analyzed using the Mann-Whitney-U-test. Statistical analyses were performed using the IBM SPSS software package (version 24).

## Figures and Tables

**Table 1:** Patient characteristics.

	Type of HEV infection		
Variable	Asymptomatic	Acute	Chronic
Patients, (%)	29 (51)	13 (23)	15 (26)
Age, years ^#,+^	40 (29–51)	51 (47–61)	58 (46–63)
Female, (%)	9 (31)	3 (23)	5 (33)
AST, U/L ^#,+^	23 (20–26)	189 (49–707)	139 (36–227)
ALT, U/L ^#,^	35 (29–49)	872 (633–2327)	137 (42–424)
Creatinine, mg/dL ^#,+^	0.9 (0.8–1)	0.9 (0.7–1.2)	1.6 (1.3–1.8)
Bilirubin, mg/dL ^#^	0.5 (0.4–0.6)	1 (0.6–2.6)	0.8 (0.5-1)
Serum IgG, g/L	10 (8.8–11.7)	10.8 (9.5–13.1)	10.3 (8.5–20.3)
Serum IgA, g/L	2.2 (1.4–3.1)	2.4 (1.6–3.8)	1.8 (1.2–2.2)
Serum IgM, g/L ^#^	0.8 (0.5–1.1)	1.3 (0.8–2.4)	1 (0.5–1.3)
Anti-HEV IgG, (%) ^#,^	13/25 (52)	8/8 (100)	10/12 (83)
Anti-HEV IgM, (%) ^#,+,^*	13/25 (52)	10/10 (100)	12/12 (100)
HEV-RNA, IU/mL ^#,+^	580 (24–3,735)	3500 (43–134,624)	740,000 (66,000–27,000,000)

Data are shown as median (IQR) or *n* (%). ^#^ Overt (acute or chronic) vs. covert (asymptomatic) HEV infection, *p* < 0.05. ^+^ Chronic vs. acute self-limiting (acute or asymptomatic) HEV infection, *p* < 0.05. * Data only in subset of individuals available. (ALT levels in acute infection referring to peak values; serological information available in 47 patients).

**Table 2:** Laboratory characteristics in patients with cryoglobulins.

Variable	Cryoglobulins	w/o Cryoglobulins	p-Value
Patients	5	52	
Age, years	55 (46–57)	49 (35–55)	0.31
Female, (%)	0 (0)	17 (33)	0.31
AST, U/L	36 (22–238)	28 (21–135)	0.63
ALT, U/L	42 (22–856)	56 (33–492)	0.69
Creatinine, mg/dL	1.6 (1.3–2.8)	0.9 (0.8–1.2)	0.007
Bilirubin, mg/dL	0.5 (0.3–1.2)	0.6 (0.4–0.9)	0.47
Serum IgG, g/L	13 (8.2–22.8)	10.3 (8.9–12.3)	0.41
Serum IgA, g/L	1.7 (1.2–7.6)	1.9 (1.5–3.2)	0.91
Serum IgM, g/L	1.6 (1.1–2.5)	0.9 (0.5–1.2)	0.024
HEV-RNA, IU/mL	670,000 (28,500–16,550,000)	2467 (140–74,500)	0.018
ANA titers *	2 (40)	11 (21)	0.58
Rheumatoid factor	0 (0)	1 (2)	1.0

w/o, without; * ANA titers of >1:160; Data is shown as median (IQR) or *n* (%).

**Table 3:** Clinical characteristics of patients with cryoglobulins.

Patient	Sex	Age	Acute/Chronic	Type of Tx	Immuno-Suppression	PLT (10^9^/L)	WBC (10^9^/L)	Lymph. (10^9^/L)
#1	M	55	Acute	-	-	155	4.3	1.36
#2	M	45	Chronic	Kidney	Tac/MMF/S	227	3.8	1.55
#3	M	56	Chronic	Heart	Eve/MMF	230	5.8	1.21
#4	M	58	Chronic	Kidney	S *	153	4.7	1.05
#5	M	46	Chronic	Heart	Eve/MMF/S	348	6.4	NA

Tx, transplantation; Tac, tacrolimus; Eve, everolimus; MMF, mycophenolate mofetil; S., steroid; PLT, platelet count; WBC, white blood cells; Lymph., lymphocyte count; NA, not available. * Patient #4 (2× kidney Tx, state after rituximab therapy due to repeated rejection) had weak immunosuppression with steroids monotherapy, due to chronic transplant nephropathy.
